# Supplementary material for: A randomized control trial feasibility evaluation of an mHealth intervention for wheelchair skill training among middle-aged and older adults
Source: PeerJ. 2017 Oct 5;5:e3879. doi: 10.7717/peerj.3879 (PMC5632536; doi:10.7717/peerj.3879)
Supplement: Supplemental Information 3 [file peerj-05-3879-s003.docx]

**OVERVIEW**

Many older adults rely on a manual wheelchair for community mobility, but are not provided with the *skills* for independent and effective use of their wheelchair. Suboptimal use of the wheelchair results in substantial social costs such as reduced engagement in meaningful activity, social isolation, and higher caregiver burden. This is a poor use of financial resources, including the cost of wheelchair acquisition and requirements for attendant care. Access to *skills training* is constrained by the expense and limited availability of skilled therapists; demands of patient and/or clinician travel; and lack of training programs designed specifically for older adults. ***E****nhancing* ***P****articipation* ***I****n the* ***C****ommunity by improving* ***Wheel****chair* ***S****kills* (EPIC WheelS) is an individualized home-training program that optimizes learning for older adults while limiting the time demands of expert trainers. Using an affordable mainstream computer tablet device, EPIC WheelS provides a structured training program that can be customized for specific users needs. The tablet is mobile, for in-chair or tabletop use, in home and community locations. A touch screen operated audio-visual display features interactive training and practice activities as well as video-recording capability. Wireless Internet enables user-trainer communication and remote program monitoring and updating by the trainer. A monitored home program that is effective and efficient for older adults has the potential for application to other target groups, particularly those in rural and remote locations with limited access to rehabilitation.

**PURPOSE:** Our purpose is to *evaluate the feasibility and effect size estimate* of a 1-month customized home training program (EPIC WheelS) for improving wheelchair mobility skills among novice older adult manual wheelchair users compared to usual care.

Despite the pervasive use of wheelchairs as an intervention in rehabilitation, the evidence for training in effective wheelchair use is still underdeveloped. *Structured* *training* has demonstrated excellent potential, but the most effective and efficient means of providing that training remains unclear. Larger multi-site clinical trials are required to establish evidence to inform and direct clinical practice. We propose that this 2-year feasibility trial is not only **critical, but also prudent, prior to moving forward** with an expensive large multi-site randomized controlled trial (RCT). Specifically the results of this study will address 1) **feasibility outcomes** (i.e., recruitment and retention; trainer and subject burden; safety; intervention administration and adherence; perceived benefit) and 2) **clinical outcomes** (i.e., effect size calculations for the primary and secondary measures). Although the principal intent is feasibility, our sample size calculation is based on the primary clinical outcome to provide a reasonable estimate of an effect size for planning the subsequent large scale RCT.

**HYPOTHESES:** We expect that the **feasibility outcomes will be sufficiently robust to support conducting a subsequent multi-site RCT.** We also expect the EPIC WheelS training program will have **a significant treatment effect** with improvement in wheelchair mobility, compared to a control group**.** The primary **clinical** endpoint will be wheelchair skill *capacity*, as measured by the Wheelchair Skills Test (WST). Secondary clinical outcomes will include wheelchair skill *safety*; confidence with wheelchair use; mobility; and user evaluation of performance in participation-oriented activities.

**BACKGROUND AND SIGNIFICANCE:**

**1. The manual wheelchair (MWC) is an important assistive device for older Canadians.**

Canada has a rapidly aging population; over the next 50 years the proportion of seniors in Canada is expected to double to more than 1 in 4.^1^ With age, the risk of a disabling health condition increases and **mobility is the most prevalent area of impairment** among older adults in Canada.^2^ The number of wheelchairs provided to address mobility issues among older adults is also rising. In 2001, an estimated 81,000 Canadians 65 years and older required a wheelchair for mobility^3^ – a rate four times the national average. A 2004 study reported that among multiple assistive device users, the MWC was considered third most important, behind eyeglasses and canes.^4^ However, the wheelchair also represents a substantial cost to consumers and the health care system. Beyond the cost of purchase, which varies from several hundred to thousands of dollars, the *process* *of assessment, procurement, fitting and delivery can reach $10,000* - these systemic costs are squandered if older adults are unable to use their wheelchair effectively to participate in important activities of life.

**2. *Without* *training* there is a much higher risk of injury, caregiver burden, social isolation and restricted participation.**

Merely acquiring a wheelchair does not guarantee independence or satisfactory performance with functional activities. In particular, environmental factors such as carpet, ramps, curbs, gravel and poor sidewalk conditions present barriers to mobility and self-propulsion. In Canada, over 90% of older MWC users experienced restricted performance in at least one major life activity^2^ (compared with 15% for those without a mobility device) and nearly 60% require assistance from a family member or other care provider for even basic mobility.^3^ Restricted mobility is associated with reduced participation and a loss in social connectedness,^5^ which can lead to feelings of isolation, stress and low self-esteem impacting overall quality of life.^6^ These challenges to independent mobility affect not only wheelchair users, but their families as well. A 2006 study of stroke survivors adjusting to wheelchair use identified substantial restriction in *caregivers’* social roles and an increased burden of care.^7^ In Canada, 25% of caregivers of the elderly are over 65 themselves,^1^ and risk both acute and overuse injury when assisting with wheelchair use.^8^ Tips and falls are common among MWC users and often result in injuries. In Canada, the **yearly** incidence of tips/falls is estimated to be 5.2%, with 4.2% resulting in injury and 2.5% requiring a visit to an Emergency Department.^9^ In the US in 2005, wheelchair-related accidents result in **1 death per week**, and treatment for a hospitalized injury is estimated at $25,000 – 75,000.^10^

Acceptance of assistive devices, such as a wheelchair, by older adults depends greatly on the adequacy of training provided, particularly during initial acquisition.^11^ Disuse, abandonment, or reluctance to use their wheelchair is a waste of personal and health-care resources, both financially and socially. Clinical studies have shown that uptake of assistive technology increases when demonstration and follow-up occur in the home.^12^ Furthermore, when provided with mobility device training in the home/community environment, older adults not only increase their usage but also better consolidate and generalize their skills and reduce their risk for injury.^13^

**3. Structured skills training is effective, but currently relies on considerable 1:1 training time.**

The Wheelchair Skills Training Program (WSTP)^14^ is the only *structured* training program reported in the literature. An expert clinician provides personal training in a clinical (hospital) setting, typically requiring 4 to 8 sessions. Several studies have demonstrated the WSTP to be safe and practical^15, 16^ and randomized controlled trials report a significant improvement in skill capacity among adult MWC users during inpatient rehabilitation^17^ and in the community^18^ [15-20% in intervention groups versus 3-8% in control groups on the WST]. More recently, a Turkish study also found comparable improvement in *safety* with skill performance.^19^ However, these studies required that either the subject travel to the training facility or the clinician travel to the home for all training. While frequent and intensive 1:1 training with an expert clinician may be optimal, therapists do not have the time or resources to offer this level of service. Therapists typically introduce wheelchair skills sporadically during rehabilitation, based on availability and immediate need. Consequently, *usual care* is highly variable and rarely involves a systematic or structured approach to training.

**4. Older adults receive little or no structured wheelchair skills training in our current system.**

While the evidence indicates structured training improves wheelchair mobility, there has not been widespread adoption within the clinical community. Even among younger populations in active rehabilitation, only 17-18% of wheelchair users receive any formal training.^20, 21^ Among those who do receive training during rehabilitation, the focus is often on indirect skills such as transferring from the wheelchair to the bed, toilet or tub. Most older adults receive **little or no training** on effective use of their MWC for daily activities. A survey of older veterans (age *m* = 65; *s* = 9) who were prescribed a wheelchair post-stroke found 53% had received *no instruction* at all on wheelchair use.^22^ In another study of veterans, more than 50% reported difficulty with even basic wheelchair propulsion, despite having access to a trained clinician and a custom-fitted wheelchair.^23^

Several factors contribute to inadequate training. *First*, many clinicians do not have sufficient knowledge of (or ability to *demonstrate*) wheelchair skills.^15^ A study of Canadian occupational therapy students found those receiving the standard curriculum had a restricted range of skills - *comparable to* *untrained MWC users.*^15^ Students who also received a structured wheelchair skills training program scored significantly higher on skill knowledge and skill performance, indicating a gap in the training capacity of many graduates. *Second*, during rehabilitation other competing demands are prioritized over wheelchair training. For patients, wheelchair training may not be perceived as relevant during their hospital stay, but becomes important later on when challenges are encountered in the community. It may be that post-discharge is a more optimal time for training. *Third*, initial wheelchair assessment and prescription typically occur during a hospital stay, but older adults are frequently discharged with a temporary wheelchair and ultimate delivery and fitting occur in the community with minimal therapist involvement and no training in mobility use. *Finally*, funding for home care and community-based services in Canada and the United States has been in decline and is insufficient to support clinician-intensive training either before or after discharge.^24, 25^ Often, the time and travel demands for both consumers and clinicians make traditional skills training cost-prohibitive.^26^ Long wait lists and the inaccessibility of rehabilitation services, particularly in rural areas, further exacerbate the problem.^26, 27^

**5. Innovative strategies to provide effective and efficient wheelchair skills training are essential.**

Delivering rehabilitation training as a monitored or self-managed home program among older adults has been effective for a variety of outcomes including strengthening,^28^ physical activity,^29^ self-care^24, 30^ and exercise.^31, 32^ Home programs are advantageous because they allow privacy for the user, occur in a familiar context, can be conveniently integrated into the users schedule, and do not require the time, effort and expense of travel.^29^ Adherence is critical to effectiveness, and a 2010 Cochrane review of exercise interventions found those that incorporated social cognitive theory (i.e., self-efficacy), were monitored, and increasingly graded complexity of the activity were more effective in improving adherence, frequency and duration of exercise.^33^ Programs are better received when they are individualized and focus on user-relevant outcomes. Interaction with peers and social support have also been identified as enhancing program adherence^28^ and provide opportunity for vicarious experience.^34^

With advances in affordability, size, portability, accessibility and user-interface simplicity, computer-related devices are becoming increasingly useful for rehabilitation interventions. Virtual reality and popular gaming systems (e.g., Nintendo Wii^TM^) have shown promising results in rehabilitation by casting therapy in a more engaging and enjoyable context. More recently, their use for physical activity training in rehabilitation among older adults has also been explored. Aarhus et al.^35^ reported a 90% participation using a commercial gaming system in a Danish nursing home, and found improvements in physical function, increased motivation and tolerance for activity, and trends towards improvement in fitness. Creating an interesting interface through the use of games is positively associated with older adults’ intention to use computer-related technologies.^35^

**OBJECTIVES**

**Primary Objectives: Feasibility Outcomes**

To assess the feasibility of study methods and procedures for a subsequent large-scale RCT, including:

1. *Process issues* of subject recruitment, consent, retention, and perceived benefit;
2. *Resource issues* of treatment adherence, burden of data collection, incorporating a health utility index, and intervention burden;
3. *Management issues* of equipment reliability, subject processing, and protocol administration; and
4. *Treatment issues* of safety, response, and treatment effect.

**Secondary Objectives: Clinical Outcomes**

1. Evaluate the effect of EPIC WheelS on wheelchair skill capacity and obtain an estimate of the treatment effect size;
2. Evaluate the effect of EPIC WheelS on wheelchair skill safety, wheelchair confidence, satisfaction with activity performance, mobility, and health-related quality of life.

**Methods**

**
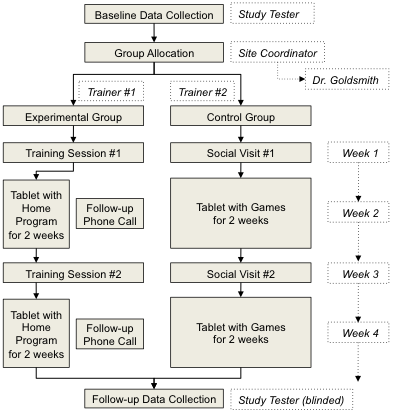
1. Research Design.** This 2-year feasibility study will use a two-site (Winnipeg and Vancouver) randomized controlled trial to compare differences in older adults’ wheelchair mobility skills with EPIC WheelS versus usual care. Subjects will be randomly assigned to the experimental group (usual care + EPIC WheelS) or a control group (usual care only) using a 1:1 allocation ratio between groups. To support balance between groups and masking of assignment, a central computerized randomization process will be designed by our team statistician (Dr. Goldsmith), with an undisclosed block size and stratified by site (Winnipeg *n* = 20; Vancouver *n* = 20). Once subjects are enrolled, the Study Tester will collect baseline data and enter them into a secure database. The Site Coordinator will contact Dr. Goldsmith via telephone or email and obtain group assignment within 48 hours. Subject contact information will be forwarded to the appropriate group Trainer to arrange for an initial training session or social visit. To address bias, subjects will be instructed not to discuss their program and Study Testers will be blinded to group allocation.

Experimental group subjects will attend 2 training sessions with an expert Trainer. The Trainer will individualize a structured home training program, delivered via a computer tablet, and subjects will train for 1 month. The Trainer will make telephone contact at the end of weeks 1 and 3. Control group subjects will receive 2 social visits to address attention balance through exposure to study personnel. In addition, they will be provided with a similar computer tablet with cognitive training games to account for tablet device and activity exposure.

**2. Assessments.** The clinical outcome measures will be collected at baseline (pre-randomization) and post-intervention. A 1-month intervention will provide ≥ 8 hours of training, which is comparable to clinical studies using the WSTP where a significant improvement in skill capacity was observed.^17-19^

**3. Subjects.** A total of 40 community-dwelling MWC users living in Winnipeg and Vancouver (20 at each site) will be recruited on a volunteer basis (see Section 8.1 for sample size justification). To optimize the impact of the treatment, individuals with less than one year of MWC use will be recruited as they are still developing routines and patterns of wheelchair use and potentially more amenable to adapting their mobility techniques.^12^

*Inclusion criteria*. Subjects will be ≥ 55 years old; live in the community within the metropolitan boundaries; self-propel a MWC ≥ 1 hour/day; use their MWC for mobility outside the home; have used a MWC for < 1 year; have a caregiver able to attend training sessions and supervise home training.

*Exclusion criteria*. Individuals will be excluded if they cannot communicate and complete study questionnaires in English; anticipate a health condition or procedure that contraindicates training (e.g., surgery scheduled which would impair physical activity); currently receive out-patient therapy that includes wheelchair mobility training; or if they exclusively foot propel their wheelchair.

**4. Outcome measures.** Wheelchair device characteristics will be collected using the Wheelchair Specification Form (see Appendix A). Descriptive characteristics including age, sex, marital status, highest level of education, primary diagnosis related to MWC use, length of time using the MWC, and propulsion method will be collected along with cognitive status measured using the Standardized Mini-Mental Status Exam (Appendix R). Handgrip strength has been demonstrated to be an accurate surrogate measure of overall strength^36^ and will be measured using a Jaymar^TM^ 5030J1 dynamometer.

**4.1 Feasibility outcomes** for process, resources, management, and treatment parameters will be measured during study administration and at study’s end. *Process* outcomes will include recruitment, consent and retention rates at each site. The Post-treatment Participant Questionnaire and follow-up interviews will be used to evaluate perceived benefit. *Resource* outcomes will be monitored by tracking study staff time logs and tablet usage data. *Management* issues will be evaluated using the trainer Evaluation Form and by tablet reliability/loss data. *Treatment* outcomes will include reporting of adverse events and statistical analysis of the treatment effect.

**4.2 Clinical outcomes** will be measured at baseline and post-treatment (1 month).

***4.2.1 Primary outcome measure: Skill Capacity***

Wheelchair mobility skill *capacity* will be measured using the Wheelchair Skills Test 4.1 (WST – See Appendix I).^14^ The WST is a structured assessment with 32 discrete mobility skills required to perform social roles in the community, each scored dichotomously as pass/fail. The WST produces a total Skill Capacity score (0-100%) reflecting the number of skills safely passed. To optimize stability of the measure, increase precision, and control for a learning effect, the WST will be administered twice at baseline and a mean baseline score calculated.^37^ Standardized equipment required for the WST (e.g., 5° & 10° ramps, gravel) is available at both test sites. The WST serves as primary outcome because it is sufficiently sensitive to detect proximal effects of the training intervention (skill capacity); it can be completed in approximately 30 minutes; and does not demonstrate floor or ceiling effects.^16^

Two systematic reviews of available wheelchair skill outcome measures confirm the WST has the strongest psychometric properties and been used most extensively in clinical trials.^38, 39^ The WST has demonstrated excellent reliability for test-retest (ICC = . 904), intra-rater (ICC = .959), and inter-rater (ICC = .968) administration.^16^ Construct validity has been supported by significant relationships with predictive variables of age, sex, MWC experience, diagnosis, and use of a lightweight wheelchair, which together accounted for 35% of variability in WST score using multiple regression (adjusted *R*^2^ = .35).^16^ Concurrent validity has been established through positive correlation with two criterion measures: therapists’ global assessment of user ability (*R*_S_ = .39 - .40) and the Functional Independence Measure (Admission score *R*_S_ = .38; Discharge score *R*_S_ = .31).^16, 40^

***4.2.2 Secondary measures***

Secondary clinical outcomes reflect more distal impacts of the intervention. Given the dearth of evidence in the literature, there is substantial value in understanding the relationship between skill acquisition and safety, confidence, community participation, mobility and utility. The secondary measures will contribute to discerning a *clinically* important impact of the EPIC WheelS intervention.

***4.2.2.1 WST Skill Safety****.* The WST also provides a total Skill Safety score (0-100%) reflecting the number of skills addressed in a safe manner, regardless of whether the skill is passed or not (see Appendix I). This is of considerable importance since training also involves learning to recognize risks and limitations. Recognition that a particular situation is unsafe or that one is not capable of safely performing an activity is vastly preferable to attempting a maneuver that may lead to accident or injury.

***4.2.2.2 Wheelchair Outcome Measure* (WhOM).** The rehabilitation literature strongly suggests that intervention outcomes should include not only externally defined measures, but also measures of *user-identified* activities of relevance and perceived satisfaction with performance.^41-43^ A 2008 review of wheelchair-specific outcomes found the WhOM was the only tool that included activities selected and weighted by the user and featured participation in social roles.^44^ Using an interview format, MWC users identify relevant activities and rate them on both Importance and Satisfaction, using an 11-point scale (0-10) (See Appendix J). The WhOM demonstrates good reliability (Test-retest ICC = 0.83-0.88; Inter-rater ICC = 0.90-0.91) and validity (correlations with LIFE-H: *r_s_* = 0.3-0.5) in use among individuals with SCI^45^ and older adults (Test-retest ICC = 0.77–1.00; correlation with QUEST *r_s_* = 0.36 -0.45).^46^ As part of the baseline data collection, the five most important activities identified with the WhOM will be used to customize the training program for experimental group subjects.

***4.2.2.3 Wheelchair Use Confidence Scale for Manual Wheelchair Users* (WheelCon-M 3.0).** Self-efficacy has been identified as a key component in the performance of wheelchair mobility skills.^47^ EPIC WheelS incorporates principles of Social Cognitive theory, including self-efficacy, to promote program adherence and skill acquisition. Preliminary research has suggested that standardized training can increase wheelchair confidence among older adults.^48^ The WheelCon is a self-report questionnaire composed of 65 statements related to confidence using a wheelchair in activities and environments, each rated on a scale from 0 (“not confident”) to 100 (“completely confident”), producing a total mean score of 0 – 100 (see Appendix K).^49^ A 2010 study identified strong test-retest reliability (ICC = 0.84) and significant correlation with comparison measures supporting its validity.^50^

***4.2.2.4 Life-Space Assessment (LSA).*** The LSA (Appendix L) will capture the mobility habits of study subjects. It is a 20-item questionnaire that tracks the locations to which wheelchair users travel on a continuum of 5 environments from the home to outside of town.^51^ Subjects report on their movement in each life-space during the past 4 weeks, the frequency of travel, and whether assistance was required. Evaluation of the LSA among power wheelchair users found excellent test-retest reliability (ICC = 0.87) with moderate to substantial concordance for 18 of 20 items (*k* = 0.47 - 0.73).^52^

***4.2.2.5 Health Utility Index Mark 3 (HUI3).*** Health utility measurement is useful in performing cost-utility and cost-effectiveness analyses of new rehabilitation interventions. The HUI3 (Appendix M) is a brief questionnaire that asks subjects about their health status, reflected in a single-score measure of *health-related quality of life* (HRQOL)*.*^53^ Test-retest reliability in a study of patients recovering from hip fracture was found to be acceptable for comparing between groups (ICC = 0.72).^54^ Differences of .03 have been found to represent meaningful change.^55^ Our study is not sufficiently powered to undertake a cost-utility analysis, but will determine the feasibility of collecting cost-utility data in a larger RCT and estimate what changes in HRQOL might be anticipated.

**5. Intervention**

***5.1 EPIC WheelS Program (experimental group)***

EPIC WheelS is a customized program combining 2 structured 1-hour sessions with an expert Trainer and 4 weeks of self-paced practice and training at home. Study Trainers will be occupational therapists with ≥ 5 years of clinical experience in wheelchair provision/training, and will have received a 2-day comprehensive orientation to the EPIC Wheels program from Mr. Giesbrecht. Subjects will attend an initial assessment/training session and receive a customized home program from their Trainer. The home program is delivered using a portable, interactive *computer tablet* that can be used for practice in their home or other community venues. After 2 weeks of practice, subjects return for a second 1-hour training session. The Trainer updates their program and they continue practicing at home for another 2 weeks. To promote adherence and safety, subjects will have a caregiver attend all training sessions and supervise their home training. The tablet collects data on *all user activity* (i.e., each touch-stroke is time-stamped), downloading usage data to the Trainer over the Internet. Trainers monitor subject activity remotely and will contact subjects by telephone at the end of weeks 1 and 3 to promote program adherence.

***5.2 Control Group***

To provide a comparable level of investigator attention, control group subjects will receive two 1-hour social visits. To control for Trainer bias, the experimental and control groups will have separate Trainers. During social visits, the Trainer will discuss subjects’ current community activities and their experience using the wheelchair, and provide verbal information related to barriers encountered (see Appendix N). Subjects will receive a computer tablet with cognitive stimulation games to account for activity and tablet device exposure. To minimize attrition, control subjects will receive a DVD with a condensed MWC skills education program after the post-intervention data collection is complete.

***5.3*** ***Usual Care (both groups)***. Both control and experimental group subjects will have received usual care for MWC mobility training during the acquisition of their wheelchair. Typical care in Canada varies between and within regions, but is typically limited to ≤ 30 minutes of training, focusing primarily on transferring from the wheelchair to the tub, toilet and bed to expedite discharge home.

**6. Training and equipment**

**6.1 Program details**


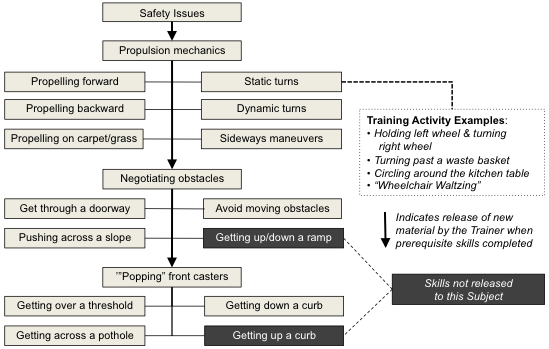
The EPIC WheelS program includes a comprehensive, structured library of educational material and training activities, organized in a hierarchy from simple to complex. Following the initial assessment and training session, Trainers will select a sub-set of skills and training activities to incorporate into a subjects’ home training program based on ability, safety, and relevance. Trainers monitor a subject’s progress through on-line access to tablet usage data and re-assessment during the second training session (@ 2 weeks), releasing more advanced skills and activities at any time via the Internet. The illustration here is an example of an individualized program, where the Trainer releases a series of skills from the EPIC WheelS library over time (Safety > Propulsion > Negotiating obstacles > Popping casters), while selectively hiding 2 skills that are not relevant for this subject.

The *home program component* is delivered using a 10” portable computer tablet (i.e. Motorola Xoom^TM^). The tablet is menu driven and interactive, using a touch-screen interface. A tablet offers considerable advantages over traditional paper/hand-out home programs. Training is provided in a multi-modal format with illustrations and videos, allowing detailed step-by-step guidance, close-ups, and demonstrations using age-appropriate older adult models. Practice activities can be clearly demonstrated (to ensure they are performed appropriately) and include imitative, function-based, and interactive game-related activities. A built-in camera enables subjects to video record their performance, which they can review for accuracy or forward to their Trainer for feedback. Subjects can also send text or voice emails to their Trainer using the tablet; Trainers will review and respond to emails within 48 hours. The tablet is housed in a rigid case with a simple strap that wraps around the wheelchair armrests. Subjects can secure the tablet on their lap and practice anywhere in their home or community, or place the tablet on another surface for viewing or video recording. Training activities are highlighted after completion, indicating to the subject they can advance to the next component. A “Progress” tab provides regular updates on the number of training sessions and minutes practiced each day, and “reward” icons are produced for meeting practice parameters to reinforce adherence. The tablet will have Internet access for email and data transfer/update capability, but the training program can operate in stand-alone mode as well. For the purposes of this study, the tablet will operate as a single-function device (i.e., other tablet applications and functions like web browsing/downloading will be disabled).

Maximizing training frequency and practice in the natural context of use are essential elements for rehabilitation home programs targeting motor skills in older adults. A 2010 Cochrane review of exercise adherence identified programs incorporating self-efficacy strategies, clinician monitoring, and progressive challenges are more effective in improving adherence, frequency, and duration of exercise.^33^ EPIC WheelS integrates principles from Social Cognitive theory^34, 56^ and strategies from Andragogy (adult learning) theory^57, 58^ as active ingredients to optimize adherence and skill acquisition. The program is individualized and progress is monitored by an expert Trainer. Skills are sequenced from simple to complex, and practice includes activities designed for a home setting. Since programs for older adults are more well-received when they focus on user-identified outcomes,^33, 59^ activities identified by the WhOM are incorporated into subjects’ programs.

The use of a computer tablet to deliver the home training program offers advantages of portability, audio-visual versatility, flexible configuration and real-time updating. Although older adults are less likely than younger people to embrace technologies such as a computer, their use of computers continues to grow. 2006 and 2010 studies in the United States found 84% of those over 60 years had experience with computers^60^ and 40% of those over 65 years are regular computer and Internet users.^61^ Use of a tablet involves some new learning, and age-related declines in memory and fluid intelligence may restrict uptake. These issues are addressed through self-paced training, structures for success experiences to build confidence, and by adapting the interface for familiarity and ease of use with minimal memory requirements.^62, 63^ Consumer, caregiver and clinician input was incorporated during EPIC WheelS program development to ensure the delivery format addresses these concerns.

**6.2 Experimental group (EPIC WheelS)**:

***6.2.1 Training Session 1.*** The Trainer will conduct the initial 1-hour session at a rehabilitation centre, following a Protocol Binder and Training Checklist (Appendix O). The training regime follows a structured sequence of skills of increasing complexity. After training, they will provide the subject and caregiver with a 30-minute orientation to the home program and tablet (including a written handbook for reference) and provide an internet-enabled tablet with the EPIC WheelS program. Subjects will be strongly encouraged to have their caregiver supervise home practice; the tablet will *prompt subjects to acknowledge review of the safety information* when performing activities with a risk for tips and falls before they can proceed. The American College of Sports Medicine best practice guidelines for older adults recommend that during physical activity, exertion remain in the moderate range and advocate use of the Borg Perceived Rate of Exertion (PRE) scale (see Appendix P).^64^ The PRE scale is rated from 6-20 with “somewhat hard” falling in the interval of 12-14.^65^ The Trainer will review the Borg PRE and guidelines for not exceeding moderate exertion during training.

***6.2.2 Home Program component***. The home program is individualized for subjects and activities are progressed by the Trainer via Internet updates to provide seamless access. A training schedule will target a minimum of 1-2 sessions/day, 15-30 minutes in length, at least 5 days per week. These guidelines are based on the National Blueprint consensus document on promoting physical activity for adults over 50 years, which advocates that lifestyle- or endurance-related activity of moderate intensity should be undertaken for at least 30 minutes (in bouts of at least 10 minutes) 5-7 days per week.^64^ Adherence with this schedule is achievable, as demonstrated by a study of home-based training program for improving hand function among stroke survivors (*n* = 77; *m* = 57 years) which obtaining 96% compliance for 1.3 hours of training per day, 7 days per week over 5 weeks.^66^ The minimum training time with EPIC WheelS (Trainer @ 2 hours + home program @ 6 hours = 8 hours) is at least comparable to 1:1 training time in other clinical studies using the WSTP.

**7. Safety**: The EPIC WheelS program incorporates extensive *safety-related material*, including teaching the safest mobility strategies; use of safety equipment; recognizing unsafe situations; and seeking assistance when skills are insufficient to address environmental barriers. At the initial training session, subjects are provided with protective wheeling gloves and a Spotter’s strap for safety. Caregivers will be invited to attend and participate in both training sessions, and subjects will be strongly encouraged to have a caregiver supervise all home training activities. Operating a wheelchair in the community carries innate risks that cannot be entirely eliminated. However, for the experimental group subjects, EPIC WheelS offers education and training designed to minimize risks of wheelchair operation. *This knowledge should reduce the risk of a fall or injury that they might be exposed to in their everyday use of a MWC had they not received this program*.

Any unsafe performance observed during training will be addressed immediately with corrective feedback. Subjects will be encouraged to contact the Study Coordinator immediately if they experience unusual discomfort, pain or physical symptoms. A Data and Safety Monitoring Board (DSMB) will review accumulating outcome data and advise the investigators regarding safety issues, evidence of benefit, and need for modification to the study design.^67^ The DSMB will include 4 members external to the research team: a statistician, a physiatrist, a clinician (OT/PT), and a MWC user. Adverse events will be documented by the study Trainer using the Treatment Protocol Checklist and will be reported to the DSMB as well as the applicable Ethics Review Board(s).

**8. Statistical analyses**

Study analyses will consider study **feasibility** as well as **clinical** (statistical) outcomes. Means and standard deviations (for continuous variables) and frequencies and proportions (for categorical variables) will be used to summarize case mix and outcome variables for groups. Qualitative comparison of variable balance will be made, including determination of prognostic importance.^68^ Descriptive statistics of training time and number of training sessions for the treatment group (collected via tablet usage data) will be used to evaluate dose-response and adherence.

**8.1 Feasibility Outcomes**

Specific feasibility objectives have been identified related to study process, resources, management, and treatment assessment.^69^ These objectives are listed below and a detailed description of each outcome and measurement variables is provided in Appendix D. Aside from the treatment outcomes that undergo statistical analysis, feasibility outcomes will be treated as binary, with “success” indicating the protocol is sufficiently robust to move forwards with the large RCT with only small or
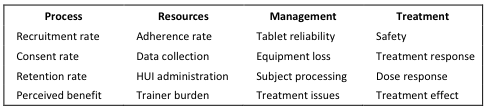
no adaptation required, and “revise” indicating a need for more substantive change before proceeding. The number and extend of objectives requiring revision will determine whether the feasibility study data can be conflated with those produced in the larger subsequent RCT.

**8.2 Clinical Outcomes**

***8.2.1 Primary analysis:*** Post-treatment WST skill *capacity* scores will be compared in the experimental and control groups using analysis of covariance (ANCOVA), controlling for baseline score as a covariate.^70^ Borm et al.^71^ demonstrate that correlation between pre- and post-intervention scores (ρ) influences analysis power; when ρ > 0.5, change score is more powerful than direct post-intervention comparison. However, when ρ lies between 0.2 and 0.8, ANCOVA (controlling for baseline score) further reduces the required sample size by 10-40% over change score. Given that preliminary data suggest ρ ~ 0.5,^72^ ANCOVA should provide the most powerful analysis, in addition to reducing error variance and allowing modification when statistical assumptions are not met.^73^ Unequal cell sizes will be accommodated using Method 1 adjustment^74^ and diagnostic assessments made for model assumptions. Significance testing (*p*) and marginal means with 95% confidence intervals will be estimated. Effect size (partial η^2^) will be calculated as a ratio of the effect and total sums of squares, with a 95% confidence interval . To preserve prognostic balance, primary analysis will be based on intention-to-treat; however, since one objective is to estimate the treatment effect, secondary analysis on a per-protocol basis (subjects who adhere to treatment) will also be conducted for comparison.^75^

***8.2.2 Secondary analysis:*** ANCOVA will be used to compare post-treatment scores between the experimental and control groups for WST skill **safety** scores as well as for wheelchair **confidence** (WheelCon) scores and **mobility** (LSA). In each case, baseline score will be used as a covariate. Subjects’ **satisfaction with performance** of meaningful activities (WhOM score) will be analyzed using analysis of variance (ANOVA). Mean administration time for the HUI will be calculated and a preliminary evaluation of change in the treatment group (paired sample *t-test*) will be conducted.

**8.3 Post-Treatment Qualitative Analysis**

After the feasibility clinical trial is complete, semi-structured interviews will be used to explore subjects’ experience with the EPIC WheelS intervention. The use of qualitative methods in conjunction with RCT studies in rehabilitation has been highly recommended to support construct validity of the quantitative outcome measures, identify additional contextual variables that mediate intervention active ingredients, and uncover unexpected findings.^76, 77^ Exploring the benefits of specific skill acquisition among EPIC WheelS subjects will provide valuable information regarding what a *clinically important difference* is for subjects. A criterion-based purposive sample (i.e., 3 highest and 3 lowest WST change score at each site) will be selected using a maximum variation strategy to provide diverse perspectives representative of the range of subjects.^78, 79^ Mr. Giesbrecht will conduct all of the individual interviews using a semi-structured question guide focusing on treatment fidelity, the training intervention, and impact on engagement in community-based activities (see Appendix F). Ed Giesbrecht has considerable clinical and research experience conducting interviews. Dr. Woodgate and Ed Giesbrecht will perform all data analysis using a *directed content analysis* approach where key concepts from existing evidence and study hypotheses form the initial coding categories.^80^ Strategies for ***authenticity and rigor*** will include sampling using treatment outcome, substantive engagement between interviewer and subjects, and documenting an audit trail. Triangulation will be supported through peer debriefing, collaborative theme analysis, member checking, and integration of the quantitative and qualitative findings.^79^ The qualitative findings will be used to enhance and interpret the clinical outcomes, such as key intervention ingredients; relevant outcomes that might not be captured; and explanation for potential conflicting quantitative results. Quantitative and qualitative findings for each subject will be summarized in table, or meta-matrix,^81^ to identify both patterns and paradoxes between the results.^82^

**8.4 Sample size justification*.*** To address the feasibility outcomes, the number of subjects is large enough to represent the target population and assess the feasibility criteria.^69^ In addition, the sample size is powered to detect a statistically significant difference between groups and provide a reasonable estimate of a treatment effect. The sample size calculations are based on the primary *clinical* outcome (WST), as this will be used in subsequent EPIC WheelS studies. Research in the field of wheelchair skills training is still maturing, with 3 published studies using actual users, and given the novel home program approach an ∝ of .10 was selected to ensure a potentially beneficial treatment effect does not go undetected (Type I error rate). To minimize the risk of identifying such an effect merely by chance (Type II error), the study is powered at 90%. Based on a sample size calculation formula for ANCOVA in RCT designs^71, 83^ each group would require 15 subjects (see Appendix Q for details). In previous Canadian trials, a 9-18% dropout rate has been reported; conservatively adjusting for a 25% dropout rate, a total study N of 40 is planned (n = 20 for each group).

Previously published studies have used younger or mixed age populations and data specific to older adults are not available. We obtained permission to use a data subset (adults > 50 years) from a yet-to-be published study^72^ providing WST change scores following training and powered our study to capture a comparable change (*m* = 9.3%; *s* = 9.5%). A difference of 9.3% corresponds to acquisition of 3 additional skills on the WST; previous studies report subjects’ perceptions of a *clinically important difference* with such improvements.^17, 18^ In fact, the skills performed on the WST are sequenced from simple (e.g. rolling forwards) to moderate (e.g., propelling on carpet) to complex (e.g., ascending 10° ramp). Research literature reporting on MWC use among older adults specifically implicates carpet, inclines, curbs, gravel and poor sidewalk conditions as barriers to independent mobility.^7^ Acquisition of **even one of these important skills** could quite reasonably represent a minimally clinically important difference (MCID) to wheelchair users, their caregivers, and those providing training. No formal MCID has been established for the WST; however, using data from a Canadian trial,^18^ a Smallest Detectable Difference calculation suggests a difference of 9.2% would exceed any measurement error or noise.^84^ A Reliability Change Index calculation indicates 3.0% is the Minimal Detectable Change required and is a reasonable proxy for MCID^84^ (details in Appendix Q).

**TIMELINE**


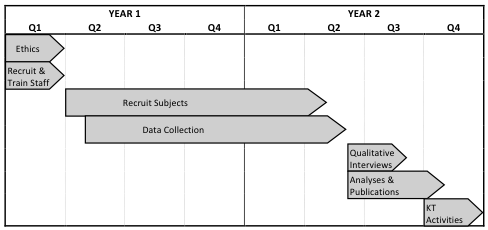
The study will be conducted over 2 years, between October 2012 and October 2014. Data collection will occur over 13 months between December 2012 and December 2013, projecting recruitment of 3 subjects per month. Qualitative interviews will then be conducted, followed by analyses and knowledge translation activities.

**FEASIBILITY OF THE STUDY**

**1. Investigator, Team members, Collaborators and Facilities.** Dr. William Miller is trained as an occupational therapist and epidemiologist. He is a research scholar who dedicates 70% of his time to his research program, which focuses on wheelchair mobility intervention and outcome measurement. He is the PI on a CIHR team grant whose mission is to improve the *power* mobility opportunities of older adults. He will be responsible for administration of the grant, oversight of the study, and site-lead for Vancouver. **Dr. Janice Eng** is trained as a physical and occupational therapist and is a BC Health Research Scholar who has extensive experience conducting multi-site randomized control trials, including an evaluation of homework-oriented rehabilitation to improve upper and lower extremity function after stroke. Dr. Eng will provide guidance with the research design and implementation. **Dr. Ian Mitchell** has training in the fields of computer science and engineering. He was instrumental in the development of the tablet software applications and will provide ongoing technology design and support for the computer tablet software application. **Dr. Charlie Goldsmith** is a clinical epidemiologist and biostatistician at Simon Fraser University who has worked extensively in musculoskeletal rehabilitation research and brings expertise in RCT design and analysis as well as cost-utility/cost-effectiveness evaluation. **Dr. Roberta Woodgate** is full professor in Nursing at the University of Manitoba. Her research focuses on the experience of disability, including quality of life and participation. She will be the Winnipeg site lead and provide expertise in collection and analysis of the post-intervention qualitative data. **Mr. Ed Giesbrecht** is an occupational therapist and a PhD candidate working with Dr. Miller, and holds a CIHR Doctoral Fellowship. He has a clinical background in MWC prescription and research experience with evaluation of wheelchair interventions, and has strong collaborative ties with the clinical community in Manitoba.

The two sites selected for the study are Winnipeg and Vancouver. These two locations represent different provincial approaches to wheelchair procurement in Canada and therefore enable us to assess whether disparate approaches to health services will contribute to complications in running a multi-site trial. Manitoba and British Columbia have substantially different wheelchair funding systems, as well as geographic, cultural and climate differences. Both sites have large populations of older adult MWC users, with representatives from both sites previously contributing to the development of the EPIC WheelS program and committed to ongoing input including knowledge translation activities. The study will be conducted at the Blusson ICORD Pavilion and Rehab Research Lab (GF Strong Rehab Hospital) in Vancouver both locations have private rooms for data collection and a separate 600 square foot space housing the WST equipment. The Health Sciences Rehabilitation Centre in Winnipeg is the primary tertiary care centre for the province and has dedicated space for wheelchair training and testing, including the WST equipment.

**2. Recruitment**. There is a substantial and growing pool of older adult MWC users to draw from. The Manitoba Wheelchair Program (MWP), a publicly funded long-term loan program and primary provincial wheelchair provider, identifies nearly ***7200 MWC users over 55*** ***years*** currently in Manitoba. Mr. Giesbrecht has established connections with clinicians and provincial agencies that provide wheelchair prescription and procurement. British Columbia has a population roughly four times as large and is expected to have at least ***25,000 older adult MWC users***. Dr. Miller has developed relationships with therapists who prescribe wheelchairs in the Lower Mainland of Vancouver. For this feasibility trial, we expect to draw on a broad pool of potential subjects through our established relationships with community-based therapists; advocacy and consumer-based groups; recruitment advertising included with delivery of wheelchairs to new users via the MWP; and public advertisement using print, television, and web-based media.

**STUDY Limitations**

Due to the nature of feasibility studies, several inherent limitations arise and will be addressed in the larger multi-site RCT study to follow. The potential for recruitment of *novice* *older adult MWC users* is unknown - this is an important feasibility outcome to address for future planning. Assessment of skill retention will not be addressed. Control group subjects may be more prone to drop-out;^85^ providing social visits, a tablet with cognitive games, and a condensed training DVD at the study conclusion will help mitigate this risk. Demonstration of a treatment effect may lack generalizability due to the restricted age group being targeted. Age is inversely related to motor skill acquisition, including wheelchair mobility skills. We anticipate that a training program proven to be effective with older adults should have at least as large a treatment effect in younger and stronger individuals; however this assumption is as yet untested. The qualitative component uses a restricted sample and is not a comprehensive exploration of subjects’ experience, but will enhance interpretation of the quantitative results and inform any fine-tuning required for the study treatment arm.

**KNOWLEDGE TRANSLATION and future directions**

Knowledge translation meetings will be held at both the Winnipeg and Vancouver sites with study stakeholders (e.g., rehabilitation clinicians, MWC users). At these meetings we will share our results and solicit feedback on the findings. This will provide an opportunity to engage these stakeholders in the subsequent large-scale RCT. We will present our results and program development to an international audience (International Seating Symposium in Nashville) and publish findings in peer reviewed journals (e.g., Archives of Physical Medicine and Rehabilitation). Most importantly, the feasibility outcomes of this study will be used to ***construct and implement a comprehensive, multi-site RCT trial that directly measures the guiding hypotheses***. This subsequent study will address skill retention using a 6-month follow-up evaluation and incorporate a yoked control group that receives a DVD demonstrating wheelchair skills but without the training and practice activity components. A large RCT will directly address evaluation of significant changes in wheelchair skill and safety; wheelchair confidence; mobility and community participation; and cost-effectiveness. After establishing its utility, the EPIC WheelS program would be made freely available. The investigators will pursue a CIHR Meetings, Planning and Dissemination Grant to disseminate the RCT findings and explore the potential of a free, downloadable training program for widespread use.

**Significance of the study**

Demonstrating the feasibility and potential treatment effect of EPIC WheelS for older adults will provide strong justification for a larger multisite RCT. There are substantial benefits for an intervention that is effective, requires only limited direct contact clinician time, and reduces demands for user travel including potential cost-savings (particularly when considering the expenses associated with wheelchair acquisition and dependence on caregivers) and improved outcomes for older adults that can be realized with minimal cost to the health care system. Future study can compare other more labour-intensive strategies (e.g., WSTP), estimate cost-effectiveness, and measure impact on community participation and caregiver burden. Evidence for the effectiveness of EPIC WheelS would inform clinical best practice and provide justification for a provincial pilot project where a single trainer coordinates service to a large group of wheelchair users across a broad geographical area. EPIC WheelS has great potential for use *across age and diagnostic groups* including training for individuals who live in rural and remote locations where access to rehabilitation programs is not practical. EPIC WheelS provides versatility as it can be delivered on multiple platforms including computers, tablets, smart phones and traditional DVD. Furthermore, the inherent capacity of the tablet device (i.e., GPS, accelerometers) can be incorporated into future EPIC WheelS versions to collect real-time user data on wheelchair performance; to create interactive training activities/games; to evaluate skill performance; and integration with other commercially available technology such as the x-box Kinect^TM^.

**References**

1. Cranswick K and Dosman D. Eldercare: What we know today. Ottawa: Statistics Canada, Catalogue no. 11-008, 2008.

2. Statistics-Canada. Participation and activity limitation survey 2006. Ottawa: 2008.

3. Shields M. Use of wheelchairs and other mobility support devices. *Health Rep*. 2004; 15: 37-41.

4. Mann WC, Llanes C, Justiss MD and Tomita M. Frail older adults' self-report of their most important assistive device. *Occup Ther J Res*. 2004; 24: 4-12.

5. Finlayson M and van Denend T. Experiencing the loss of mobility: Perspectives of older adults with MS. *Disabil Rehabil*. 2003; 25: 1168-80.

6. Simpson RC, LoPresti EF and Cooper RA. How many people would benefit from a smart wheelchair? *J Rehabil Res Develop*. 2008; 45: 53-72.

7. Laliberte Rudman D, Hebert D and Reid D. Living in a restricted occupational world: The

occupational experiences of stroke survivors who are wheelchair users and their caregivers. *Can J*

*Occup Ther*. 2006; 73: 141-52.

8. Kirby RL, Mifflen NJ, Thibault DL, et al. The manual wheelchair-handling skills of caregivers and the effect of training. *Arch Phys Med Rehabil*. 2004; 85: 2011-9.

9. Kirby RL, Ackroyd-Stolarz SA, Brown MG, Kirkland SA and MacLeod DA. Wheelchair-related

accidents caused by tips and falls among noninstitutionalized users of manually propelled wheelchairs in Nova Scotia. *Am J Phys Med Rehabil*. 1994; 73: 319-30.

10. Gavin-Dreschnack D, Nelson A, Fitzgerald S, et al. Wheelchair-related falls: Current evidence and directions for improved quality care. *J Nurs Care Qual*. 2005; 20: 119-27.

11. Kraskowsky LH and Finlayson M. Factors affecting older adults' use of adaptive equipment: Review of the literature. *Am J Occup Ther*. 2001; 55: 303-10.

12. Gitlin LN and Levine RE. Prescribing adaptive devics to the elderly: Principles for treatment in the home. *Int J Technol Aging*. 1992; 5: 107-20.

13. Walker KA, Morgan KA, Morris CL, et al. Development of a community mobility skills course for people who use mobility devices. *Am J Occup Ther*. 2010; 64: 547-54.

14. Kirby RL. Wheelchair Skills Program Version 4.1. 2011. www.wheelchairskillsprogram.ca

15. Coolen AL, Kirby RL, Landry J, et al. Wheelchair skills training program for clinicians: A randomized controlled trial with occupational therapy students. *Arch Phys Med Rehabil*. 2004; 85: 1160-7.

16. Kirby RL, Dupuis DJ, Macphee AH, et al. The wheelchair skills test (version 2.4): measurement properties. *Arch Phys Med Rehabil*. 2004; 85: 794-804.

17. MacPhee AH, Kirby RL, Coolen AL, et al. Wheelchair skills training program: A randomized clinical trial of wheelchair users undergoing initial rehabilitation. *Arch Phys Med Rehabil*. 2004; 85: 41-50.

18. Best KL, Kirby RL, Smith C and MacLeod DA. Wheelchair skills training for community-based manual wheelchair users: A randomized controlled trial. *Arch Phys Med Rehabil*. 2005; 86: 2316-23.

19. Ozturk A and Ucsular FD. Effectiveness of a wheelchair skills training programme for community-living users of manual wheelchairs in Turkey: A randomized controlled trial. *Clin Rehabil*. 2011; 25: 416-24.

20. Karmarkar AM, Collins DM, Kelleher A, et al. Manual wheelchair-related mobility characteristics of older adults in nursing homes. *Disabil Rehabil Assist Technol*. 2010; 5: 428-37.

21. Smith C and Kirby RL. Manual wheelchair skills capacity and safety of residents of a long-term care facility. *Arch Phys Med Rehabil*. 2011; 92: 663-9.

22. Garber SL, Bunzel R and Monga TN. Wheelchair utilization and satisfaction following cerebral vascular accident. *J Rehabil Res Dev*. 2002; 39: 521-34.

23. Ganesh S, Hayter A, Kim J, et al. Wheelchair use by veterans newly prescribed a manual wheelchair. *Arch Phys Med Rehabil*. 2007; 88: 434-9.

24. Gill TM, Baker DI, Gottschalk M, et al. A program to prevent functional decline in physically frail, elderly persons who live at home. *N Engl J Med*. 2002; 347: 1068-74.

25. Tousignant M, Dubuc N, Hebert R and Coulombe C. Home-care programmes for older adults with disabilities in Canada: how can we assess the adequacy of services provided compared with the needs of users? *Health Soc Care Community*. 2007; 15: 1-7.

26. Sanford JA, Griffiths PC, Richardson P, et al. The effects of in-home rehabilitation on task self efficacy in mobility-impaired adults: A randomized clinical trial. *J Am Geriatr Soc*. 2006; 54: 1641-8.

27. Tousignant M, Boissy P, Corriveau H and Moffet H. In home telerehabilitation for older adults after discharge from an acute hospital or rehabilitation unit: A proof-of-concept study and costs estimation. *Disabil Rehabil Assist Technol*. 2006; 1: 209-16.

28. Layne JE, Sampson SE, Mallio CJ, et al. Successful dissemination of a community-based strength training program for older adults by peer and professional leaders: The people exercising program. *J Am Geriatr Soc*. 2008; 56: 2323-9.

29. Jette AM, Rooks D, Lachman M, et al. Home-based resistance training: Predictors of participation and adherence. *Gerontologist*. 1998; 38: 412-21.

30. Hoenig H, Sanford JA, Butterfield T, et al. Development of a teletechnology protocol for inhome rehabilitation. *J Rehabil Res Develop*. 2006; 43: 287-98.

31. Campbell AJ, Robertson MC, Gardner MM, et al. Randomised controlled trial of a general practice programme of home based exercise to prevent falls in elderly women. *BMJ*. 1997; 315: 1065-9.

32. Tinetti ME, Baker DI, McAvay G, et al. A multifactorial intervention to reduce the risk of falling among elderly people living in the community. *N Engl J Med*. 1994; 331: 821-7.

33. Jordan JL, Holden MA, Mason EE and Foster NE. Interventions to improve adherence to exercise for chronic musculoskeletal pain in adults. *Cochrane Database Syst Rev*. 2010: CD005956.

34. Bandura A. *Self-efficacy: The exercise of control*. New York: W. H. Freeman and Company, 1997.

35. Aarhus R, Gronvall E, Larsen SB and Wollsen S. Turning training into play: Embodied gaming, seniors, physical training and motivation. *Gerontechnology*. 2011; 10: 110-20.

36. Bohannon RW. Hand-grip dynamometry predicts future outcomes in aging adults. *J Geriatr Phys Ther*. 2008; 31: 3-10.

37. Friedman LM, Furberg CD and DeMets DL. *Fundamentals of clinical trials*. 4th ed. New York: Springer Science+Business Media, 2010.

38. Fliess-Douer O, Vanlandewijck YC, Lubel Manor G and Van Der Woude LH. A systematic review of wheelchair skills tests for manual wheelchair users with a spinal cord injury: Towards a standardized outcome measure. *Clin Rehabil*. 2010; 24: 867-86.

39. Kilkens OJ, Post MW, Dallmeijer AJ, Seelen HA and van der Woude LH. Wheelchair skills tests: A systematic review. *Clin Rehabil*. 2003; 17: 418-30

40. Kirby RL, Swuste J, Dupuis DJ, MacLeod DA a\ d Monroe R. The Wheelchair Skills Test: A pilot study of a new outcome measure. *Arch Phys Med Rehabil*. 2002; 83: 10-8.

41. Hammel J, Magasi S, Heinemann A, Whiteneck G, Bogner J and Rodriguez E. What does participation mean? An insider perspective from people with disabilities. *Disabil Rehabil*. 2008; 30:1445-60.

42. Hoenig H, Giacobbi P and Levy CE. Methodological challenges confronting researchers of wheeled mobility aids and other assistive technologies. *Disabil Rehabil Assist Technol*. 2007; 2: 159-68.

43. Mallinson T and Fischer H. Rehabilitation research. *Am J Occup Ther*. 2010; 64: 506-14.

44. Mortenson WB, Miller WC and Auger C. Issues for the selection of wheelchair-specific activity and participation outcome measures: A review. *Arch Phys Med Rehabil*. 2008; 89: 1177-86.

45. Miller WC, Garden J and Mortenson WB. Measurement properties of the wheelchair outcome measure in individuals with spinal cord injury. *Spinal Cord*. 2011; 49: 995-1000.

46. Auger C, Demers L, Gelinas I, et al. Reliability and validity of the telephone administration of the wheelchair outcome measure (WhOM) for middle-aged and older users of power mobility devices. *J Rehabil Med*. 2010; 42: 574-81.

47. Rushton PW and Miller WC. Development of an assessment to measure self-efficacy with wheelchair mobility. Paper presented at the *Canadian Association of Occupational Therapists Annual* *Conference*. Ottawa, ON, 2009.

48. Sakakibara B, Miller WC, Souza M and Nikolova V. Efficacy of the Wheelchiar Skills Training Program to improve confidence with using a mnaual wheelchair. Poster presented at the *Festival of* *International Conferences on Caregiving, Diability, Aging and Technology*. Toronto, ON, 2011.

49. Rushton PW, Miller WC, Lee Kirby R, Eng JJ and Yip J. Development and content validation of the Wheelchair Use Confidence Scale: a mixed-methods study. *Disabil Rehabil Assist Technol*. 2011; 6: 57-66.

50. Rushton PW, Miller WC, Mortenson WB and Garden J. Satisfaction with participation using a manual wheelchair among individuals with spinal cord injury. *Spinal Cord*. 2010; 48: 691-6.

51. Auger C, Demers L, Gelinas I, et al. Life-space mobility of middle-aged and older adults at various stages of usage of power mobility devices. *Arch Phys Med Rehabil*. 2010; 91: 765-73.

52. Auger C, Demers L, Gelinas I, et al. Development of a French-Canadian version of the Life- Space Assessment (LSA-F): content validity, reliability and applicability for power mobility device users. *Disabil Rehabil Assist Technol*. 2009; 4: 31-41.

53. Torrance GW, Furlong W and Feeny D. Health utility estimation. *Expert Rev Pharmacoecon Outcomes Res*. 2002; 2: 99-108.

54. Jones CA, Feeny D and Eng K. Test-retest reliability of health utilities index scores: Evidence from hip fracture. *Int J Technol Assess Health Care*. 2005; 21: 393-8.

55. Drummond M. Introducing economic and quality of life measurements into clinical studies. *Ann Med*. 2001; 33: 344-9.

56. Bandura A. *Social foundations of thought and action*. Englewood Cliffs, NJ: Prentice Hall, 1986.57. Knowles MS. *The modern practice of adult education: From Pedagogy to Andragogy*. New York: Cambridge, The Adult Education Company, 1980.

58. Merriam SB. Andragogy and self-directed learning: Pillars of adult learning theory. *New Directions for Adult and Continuing Educaiton*. 2001; 89: 3-13.

59. Shaughnessy M and Resnick BM. Using theory to develop an exercise intervention for patients post stroke. *Top Stroke Rehabil*. 2009; 16: 140-6.

60. Czaja SJ, Charness N, Fisk AD, et al. Factors predicting the use of technology: findings from the Center for Research and Education on Aging and Technology Enhancement (CREATE). *Psychol Aging*. 2006; 21: 333-52.

61. Charness N, Fox MC and Mitchum AL. Lifespan cognition and information technology. In: Fingerman K, Berg C, Antonnuci T and Smith J, (eds.). *Handbook of lifespan psychology*. New York: Springer, 2010.

62. Callahan JS, Kiker DS and Cross T. Does method matter? A meta-analysis of the effects of training method on older learner training performance. *J Manag*. 2003; 29.

63. Jay GM and Willis SL. Influence of direct coputer experience on older adults' attitude toward computers. *J Gerontol Psychol Sci*. 1992; 47: P250-7.

64. Cress ME, Buchner DM, Prohaska T, et al. Best practices for physical activity programs and behavior counseling in older adult populations. *J Aging Phys Act*. 2005; 13: 61-74.

65. Borg G. *Borg's perceived exertion and pain scales*. Champaign, IL: Human Kinetics, 1998.

66. Alon G, Sunnerhagen KS, Geurts AC and Ohry A. A home-based, self-administered stimulation program to improve selected hand functions of chronic stroke. *NeuroRehabil*. 2003; 18: 215-25.

67. DeMets DL. Data and safety monitoring boards. In: Armitage P and Colton T, (eds.). *Encyclopedia of biostatistics*. Chichester: John Wiley & Sons, 1998, p. 1066-71.

68. Altman DG. Comparability of randomised groups. *Statistician*. 1985; 34: 125-36.

69. Thabane L, Ma J, Chu R, et al. A tutorial on pilot studies: the what, why and how. *BMC Med Res Methodol*. 2010; 10: 1.

70. Tabachnick BG and Fidell LS. *Using multivariate statistics*. 5th ed. Boston: Pearson Education, Inc, 2007.

71. Borm GF, Fransen J and Lemmens WA. A simple sample size formula for analysis of covariance in randomized clinical trials. *J Clin Epidemiol*. 2007; 60: 1234-8.

72. Routhier F. Centre interdisciplinaire de recherche en réadaptation et intégration sociale (CIRRIS), Laval, PQ. Personal Communication, 2011.

73. Dimitrov DM and Rumrill PD, Jr. Pretest-posttest designs and measurement of change. *Work*. 2003; 20: 159-65.

74. Overall JE and Spiegel DK. Concerning least squares analysis of experimental data. *Psychol Bull*. 1969; 72: 311-22.

75. Moncur R and Larmer J. Clinical applicability of intention-to-treat analyses. *McMaster Univ Med J*. 2009; 6: 39-41.

76. Campbell NC, Murray E, Darbyshire J, et al. Designing and evaluating complex interventions to improve health care. *BMJ*. 2007; 334: 455-9.

77. Rauscher L and Greenfield BH. Advancements in contemporary physical therapy research: use of mixed methods designs. *Phys Ther*. 2009; 89: 91-100.

78. Kroll T, Neri MT and Miller K. Using mixed methods in disability and rehabilitation research. *Rehabil Nurs*. 2005; 30: 106-13; discussion 13.

79. Ohman A. Qualitative methodology for rehabilitation research. *J Rehabil Med*. 2005; 37: 273-80. Hsieh HF and Shannon SE. Three approaches to qualitative content analysis. *Qual Health Res*. 2005; 15: 1277-88.

81. Miles MB and Huberman AM. *Qualitative data anaylsis*. 2nd ed. Thousand Oaks, CA: SAGE Publications, 1994.

82. O'Cathain A, Murphy E and Nicholl J. Three techniques for integrating data in mixed methods studies. *BMJ*. 2010; 341: c4587.

83. McDonald JH. Analysis of covariance. In: McDonald, J.H. 2009. Handbook of Biological Statistics. Sparky House Publishing, Baltimore., 2009.

84. Beaton DE, Boers M and Wells GA. Many faces of the minimal clinically important difference (MCID): a literature review and directions for future research. *Curr Opin Rheumatol*. 2002; 14: 109-14.

85. Whyte J and Hart T. It's more than a black box; it's a Russian doll: defining rehabilitation treatments. *Am J Phys Med Rehabil*. 2003; 82: 639-52.

86. Mendoza, R. J., Pittenger, D. J., Saftler Savage, F., & Weinstein, C. S. (2003). A protocol for assessment of risk in wheelchair driving within a healthcare facility. *Disability and Rehabilitation, 25*(10), 520-526.
